# Publishing negative results is good for science

**DOI:** 10.1099/acmi.0.000792

**Published:** 2024-04-02

**Authors:** Elisabeth M. Bik

**Affiliations:** 1 Science Integrity Consultant at Harbers Bik LLC, San Francisco, CA, USA

**Keywords:** negative outcomes, null results, open science, reproducibility, science integrity

## Abstract

Scientists face challenges in publishing negative results, because most scientific journals are biassed in accepting positive and novel findings. Despite their importance, negative results often go unpublished, leading to duplication of efforts, biassed meta-analyses, and ethical concerns regarding animal and human studies. In this light, the initiative by *Access Microbiology* to collect and publish negative results in the field of microbiology is a very important and valuable contribution towards unbiassed science.

Science is hard. It can take years to find the right conditions to grow a particular micro-organism, to discover the specific components to make an experiment work, or to understand the many enzymes and conditions of a particular pathway. Often, experiments do not go as planned. Previous research cannot be reproduced, a drug that was promising in cell culture does not work in an animal model, or the obtained data might not fit an attractive hypothesis. Research notebooks and laboratory basements are filled with negative results.

That does not mean that such negative findings are without value. Because researchers base their experiments on the work that others have published previously, it is very valuable to not only know which experiments worked, but also which ones did not work. It would save researchers a lot of time if they would know what others had already tried, so they can focus on alternative approaches.

But publishing negative results is hard. Most journals are specifically looking for data that is novel, attractive and news-worthy. Replication studies or negative results might be very valuable to share with other scientists, but are hard to get published. Editors do not expect those to be read and cited often, and so those type of studies are often desk rejected and remain unpublished, leading to a publication bias of positive studies [1].

At the same time, there is an increased pressure on scientists to publish. The current community likes to focus on easy-to-measure metrics, such as numbers of papers a scientist has published, the impact factors of the journals those papers has been published in, or the numbers of times those papers have been cited. Such metrics do not necessarily measure the quality of a researcher, but they are easy to apply if, e.g. selecting a candidate for a tenure position out of a stack of hundreds of applicants.

The narrow focus on publishing positive and appealing data, combined with an increased pressure to publish has led some researchers to cut corners – or worse. If the experiments do not result in positive outcomes to publish, people might feel tempted to cheat. Not because they started a career in science with the intention to do fraudulent science. But because they feel pressured by the strict requirements of their university to publish several papers before they can obtain their PhD or MD or become tenured, or by a bullying principal investigator (PI) who threatens to fire them unless they give the PI the desired data. The damage to science is huge. Scientific paper mill scammers and citation cartels have infiltrated the scientific literature by producing massive amounts of papers with low quality and fake data [2]. It is currently estimated that about 2 % of all scientific papers might be paper mill articles.

Allowing researchers to be able to publish negative results would serve science in several ways [3].

First, it prevents duplicative efforts, where researchers might test a hypothesis that has been proven wrong previously by other researchers, but where those results had not been published. Making negative results available for other researchers would save considerable research effort and money.

Secondly, the publication of negative outcomes will result in less bias in future meta-analysis studies, which are likely to have incorrect conclusions if negative results are not included because they were never published. A less-biassed range of outcomes will ensure such meta-analyses are much more valuable.

Then, in the case of animal experiments or studies involving human participants, it would be unethical to not publish those data. Such studies might involve the loss of animal lives or human patients participating in potential risky studies, and researchers have a moral obligation to publish the analysis of those studies, independent of the outcome. Preregistration of clinical trials has been a great incentive for negative outcomes to be published, but efforts in animal studies are lagging behind. A similar argument could be made for studies funded with taxpayers' money or through donations. Even null results deserve to be published.

In addition, the publication of negative results would also promote replication and reproducibility efforts. If researchers know that their replication efforts will be publishable, even if the outcome is that a study was not reproducible in their hands, it might encourage more of such studies. It might also bring to light missing details in the methods that turn out to be important.

Furthermore, it rewards scientists for rigour and effort, where months or years of their work will be publishable even if the outcome is negative. This will help science to focus more on quality than on quantity.

And finally, the ability to publish negative results will lead to increased integrity of scientific studies, where scientists might not feel the pressure to falsify or fabricate data to make their results look ‘better’.

Null results are a key part of sound science and deserve to be published. In this light, *Access Microbiology*’s new collection of negative results is a fantastic initiative. Since the platform’s launch in 2019, they have published several impactful microbiology null studies and this collection will continue to expand as new negative-result papers are published. I applaud the Microbiology Society for supporting this platform and hope that many other journals and societies will follow in their footsteps. Science is hard, but it should be unbiassed.

## Abbreviation

PI: principal investigator

## References

[1] Fanelli D (2012). Negative results are disappearing from most disciplines and countries. Scientometrics.

[2] Van Noorden R (2023). How big is science’s fake-paper problem?. Nature.

[3] Mlinarić A, Horvat M, Šupak Smolčić V (2017). Dealing with the positive publication bias: Why you should really publish your negative results. Biochem Med.

